# Population-Based Passive Tick Surveillance and Detection of Expanding Foci of Blacklegged Ticks *Ixodes scapularis* and the Lyme Disease Agent *Borrelia burgdorferi* in Ontario, Canada

**DOI:** 10.1371/journal.pone.0105358

**Published:** 2014-08-29

**Authors:** Mark P. Nelder, Curtis Russell, L. Robbin Lindsay, Badal Dhar, Samir N. Patel, Steven Johnson, Stephen Moore, Erik Kristjanson, Ye Li, Filip Ralevski

**Affiliations:** 1 Enteric, Zoonotic and Vector-Borne Diseases, Communicable and Infectious Disease Prevention and Control, Public Health Ontario, Toronto, Ontario, Canada; 2 Field Studies, Zoonotic Diseases and Special Pathogens, National Microbiology Laboratory, Public Health Agency of Canada, Winnipeg, Manitoba, Canada; 3 Analytic Services, Knowledge Services, Public Health Ontario, Toronto, Ontario; 4 Public Health Ontario Laboratory - Toronto, Public Health Ontario, Toronto, Ontario; 5 Department of Laboratory Medicine and Pathobiology, University of Toronto, Toronto, Ontario; 6 Dalla Lana School of Public Health, Toronto, Ontario; Metabiota, United States of America

## Abstract

We identified ticks submitted by the public from 2008 through 2012 in Ontario, Canada, and tested blacklegged ticks *Ixodes scapularis* for *Borrelia burgdorferi* and *Anaplasma phagocytophilum*. Among the 18 species of ticks identified, *I*. *scapularis*, *Dermacentor variabilis*, *Ixodes cookei* and *Amblyomma americanum* represented 98.1% of the 14,369 ticks submitted. Rates of blacklegged tick submission per 100,000 population were highest in Ontario's Eastern region; *D*. *variabilis* in Central West and Eastern regions; *I. cookei* in Eastern and South West regions; and *A*. *americanum* had a scattered distribution. Rates of blacklegged tick submission per 100,000 population were highest from children (0–9 years old) and older adults (55–74 years old). In two health units in the Eastern region (i.e., Leeds, Grenville & Lanark District and Kingston-Frontenac and Lennox & Addington), the rate of submission for engorged and *B*. *burgdorferi*-positive blacklegged ticks was 47× higher than the rest of Ontario. Rate of spread for blacklegged ticks was relatively faster and across a larger geographic area along the northern shore of Lake Ontario/St. Lawrence River, compared with slower spread from isolated populations along the northern shore of Lake Erie. The infection prevalence of *B*. *burgdorferi* in blacklegged ticks increased in Ontario over the study period from 8.4% in 2008 to 19.1% in 2012. The prevalence of *B*. *burgdorferi*-positive blacklegged ticks increased yearly during the surveillance period and, while increases were not uniform across all regions, increases were greatest in the Central West region, followed by Eastern and South West regions. The overall infection prevalence of *A*. *phagocytophilum* in blacklegged ticks was 0.3%. This study provides essential information on ticks of medical importance in Ontario, and identifies demographic and geographic areas for focused public education on the prevention of tick bites and tick-borne diseases.

## Introduction

*Borrelia burgdorferi* sensu stricto, the agent of Lyme disease, has emerged since the mid-1970s to become the most common vector-borne human pathogen in the USA; however, the emergence has lagged in southern Canada [1], [2]. Researchers attribute the delayed occurrence of Lyme disease in Ontario and elsewhere in southern Canada to the limited distribution of the primary vector, the blacklegged tick *Ixodes scapularis*; but from the 1970s through the 1990s, the range of the blacklegged tick gradually expanded northward from the Northeastern and Midwestern USA [3]–[5]. Increases in the mean annual degree days above 0°C is considered the primary factor responsible for the northward expansion of blacklegged tick populations into southern Canada [3], [6]. In the early 1970s, Watson and Anderson reported on the first population of blacklegged ticks in Canada at Long Point Provincial Park, Ontario, along the northern shore of Lake Erie [7]. Beginning in the mid-1990s and through the 2000s, additional established populations of blacklegged ticks were detected along the northern shores of Lake Erie (i.e., Point Pelee National Park, Rondeau Provincial Park, Turkey Point Provincial Park and the Wainfleet Conservation Area), Lake Ontario (Prince Edward Point National Wildlife Area) and the St. Lawrence River (St. Lawrence Islands National Park) [2], [8]–[10]. Passive tick surveillance, through this period of establishment, detected blacklegged ticks more frequently in localities outside of these established populations.

Since the late 1960s, public health authorities in Ontario have used passive tick surveillance to provide species identifications to the public and health care professionals and to assess the risk of tick-borne diseases. Ticks submitted for identification to the provincial public health laboratory from 1967 through 1977 did not include the blacklegged tick; rather *Dermacentor variabilis* and *Ixodes cookei* dominated submissions [11]. In response to discoveries of *B*. *burgdorferi*-infected blacklegged ticks in southern Ontario in 1987, a national passive tick surveillance program began in 1990 focusing on blacklegged ticks, whereby ticks were submitted from the public through public health authorities and health care professionals for identification and pathogen detection [12], [13]. Here we investigate ticks submitted for identification and testing from 2008 through 2012 in Ontario, Canada. The objective of this work is to determine: 1) the species of ticks found on humans in Ontario and to discuss the vector potential of the most abundant species, 2) the geographic and seasonal distribution patterns of these ticks, 3) the sex and age of persons submitting these ticks and 4) the patterns of geographic distribution and incidence of blacklegged ticks infected with *B. burgdorferi* and *Anaplasma phagocytophilum*.

## Materials and Methods

### Study Location and Ethics Statement

**Figure 1:**
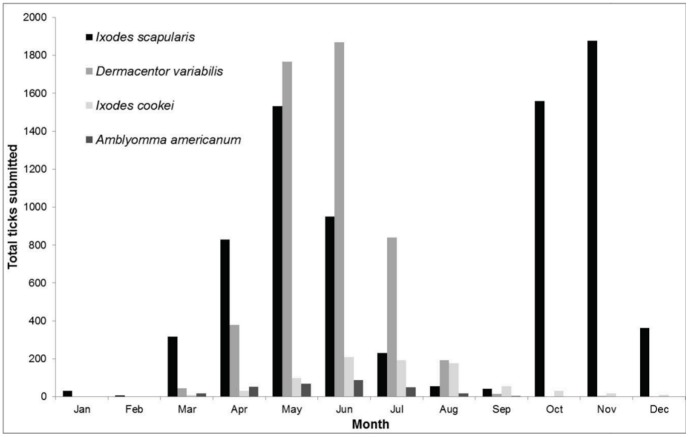
Seasonality of adult ticks submitted in Ontario, Canada (2008–2012).

Ontario, Canada is located in the Great Lakes region of North America (42.0 to 56.8° N, −74.4 to −95.2° W). Ontario is the most populous province in Canada (12.8 million in 2006) and occupies greater than 900,000 km^2^ [14]. Ontario's population is concentrated in Southern Ontario, an area dominated by a moderate, humid, continental climate with a mixture of agricultural, deciduous forest and urban landscapes [15]. In Ontario, 36 public health units administer public health services including aspects of the passive tick surveillance program (Figure 1A provides a list of public health units and their respective three-letter abbreviations). Public health units are further organized into seven health regions: Central East (DUR, HKP, PEE, PTC, SMD, YRK), Central West (BRN, HAL, HAM, HDN, NIA, WAT, WDG), Eastern (EOH, HPE, KFL, LGL, OTT, REN), North East (ALG, NPS, PQP, SUD, TSK), North West (NWR, THB), South West (CHK, ELG, GBO, HUR, LAM, MSL, OXF, PDH, WEC) and Toronto (TOR) (Figure S1).

This manuscript reports on surveillance activities, and therefore research ethics committee approval was not required. Public Health Ontario (PHO) has a legislated mandate “*to develop, collect, use, analyse and disclose data, including population health, surveillance and epidemiological data, across sectors, including human health…in a manner that informs and enhances healthy public policy and public health planning, evaluation and action*” (Ontario Agency for Health Protection and Promotion Act, SO 2007, c 10. Available at: http://www.e-laws.gov.on.ca/html/statutes/english/elaws_statutes_07o10_e.htm). The collection, use, analysis, and disclosure of data described in the current manuscript fall entirely within this surveillance mandate. No additional data were collected, and no data outside of our mandated jurisdiction were included. The data we used never contained personal identifiers. Essentially, no-de-identification occurred as tick submissions are individually identified based on a unique ID number.

### Passive Surveillance System

The passive surveillance system in Ontario is a dual system, in which two complementary programs provide data on Ontario ticks. The Public Health Ontario Laboratory (PHOL; Toronto, Ontario) performs one component, focusing on tick species identification. The National Microbiology Laboratory (NML, Public Health Agency Canada; Winnipeg, Manitoba) performs the complementary component, focusing on testing blacklegged ticks for *B*. *burgdorferi* and *A*. *phagocytophilum*. The public submits ticks through health care professionals (clinics, hospitals) or public health units. Ticks identified by PHOL as blacklegged ticks are forwarded to NML for pathogen detection. Pertinent data captured by the PHOL program include the submitter's city of residence, age and sex; date of submission; and species of tick identified. In the fall of 2009, PHOL sent a letter to public health units and health care professionals indicating that we would no longer accept ticks from non-human animals for identification or further testing. Although this change in policy meant a reduction in ticks from non-human animals, most of the results presented here are based on ticks submitted by human hosts only unless otherwise noted.

The core elements of the passive surveillance system developed at the NML are similar to those used at PHOL and were described previously [13]. The primary differences between the two surveillance systems is that NML asks specifically for information about the locality of tick acquisition (and not just residence of submitter) and submitter's travel history, while recording the tick's engorgement status, life-cycle stage and pathogen test results. Ticks in this report were either unengorged (no evidence of a blood meal) or engorged (i.e., slightly, partially or fully engorged). The engorged categories included ticks that were considered to have fed a sufficient duration to allow for transmission of *B*. *burgdorferi* (>24 hours).

Promotion of the passive surveillance system differs among health units, based on the presence or absence of blacklegged ticks. Because of expanding tick populations or increased incidence of Lyme disease, certain health units (e.g., KFL, LGL) have intensified their public outreach efforts. Consequently, we expected a degree of bias because of increased submissions of all tick species from health units that have emphasized tick submissions via the passive surveillance system. A member of public from whom a tick was collected, or a person for whom a submission was made on their behalf (e.g., a parent collects and submits tick from their child), is considered a submitter.

### Detection of *B. burgdorferi* and *A. phagocytophilum*

Blacklegged ticks were tested for the presence of *B*. *burgdorferi* and *A*. *phagocytophilum* by using real-time PCR as previously described [13], [16]. Ticks were usually tested singly; however, when multiple ticks were submitted from the same person, then pools of ticks were tested. Briefly, DNA was extracted from ticks using QIAGEN DNeasy 96 tissue kits (QIAGEN Inc., Mississauga, ON, Canada) optimized for recovery of low-copy number DNA from ticks, and extraction efficiency was assessed using primers specific for the tick 16S rRNA gene [13]. DNA extracts were screened for evidence of *B*. *burgdorferi* and *A*. *phagocytophilum* infection by using a multiplex real-time PCR targeting their 23S rRNA and msp2 genes, respectively, as previously described [17]. *Borrelia burgdorferi* DNA was then confirmed in positive samples by using primers and a probe targeting the OspA gene [13]. Samples that were positive for *A*. *phagocytophilum* by using the msp2 gene targets were confirmed, using primers for the 16S rRNA gene [16].

### Descriptive Analyses

Variable means (e.g., age of submitters of different tick species) were analyzed by using one-way analysis of variance and significant differences among means were determined by Tukey's method of multiple comparisons, with statistical tests considered significant at a familywise error rate of p<0.05 (R, version 3.1.0, The R Project for Statistical Computing) [18]. We used Pearson product moment correlations to test independence of variables (e.g., number ticks submitted per age group and rate of submission per age group). In addition, we used Chi-square analyses to test the null hypothesis that there is no significant difference between the expected and observed numbers of men and women submitters for a particular tick species. The cumulative number of towns from which a blacklegged tick was submitted during the surveillance period was examined using linear regression with Time (in months since January 2008) and Region (the combined health units of EOH, HPE, KFL and LGL along the northern shore of Lake Ontario/St. Lawrence River and the combined health units of CHK, ELG, HDN, NIA and WEC along the northern shore of Lake Erie) as covariates in the model. The interaction between Time and Region is also included to examine if the rates of spread are different between these two regions. Data is available upon request via PHO at http://www.publichealthontario.ca/en/About/Pages/privacy.aspx.

### Mapping

Available towns, cities and place names were geocoded to provide latitude and longitude values by using ArcMap v10.1 (ESRI, Redlands, California, USA) and Google Maps (Google, Mountain View, CA, USA). We spatially aggregated point locations (with corresponding tick data) to the 2006 census year CSD level for mapping [19]. We used Natural Breaks (Jenks) or manual classification methods to determine map classes. All maps, summary tables and geoprocessing tasks were created or performed by using ESRI's ArcGIS v10.1 software. Rates of tick submission were generated by using 2006 population data for Ontario health units or census-subdivisions (CSD) [14], [19].

### Multivariable Analyses

We modelled blacklegged tick counts using a Generalized Additive Model (GAM) with a Poisson response for the number of blacklegged ticks submitted as the dependent variable, including Year (linear) and Region (categorical) as well as their interaction term to see if the differences among regions vary by year. In addition, we included month (1–12) as a smooth term to model the non-linear trend of the outcome within a year. Region was defined as North West, North East, Eastern, Central East, Central West and South West (previously described). North East was chosen as the reference region since it had the lowest relative abundance of blacklegged ticks.

We also modelled the increase in *B. burgdorferi* and *A. phagocytophilum*-positive blacklegged ticks using a logistic regression in a similar fashion, with the test result (positive or negative) of *B. burgdorferi* or *A. phagocytophilum* blacklegged ticks as the response variable, Year and Region as covariates and Month as a smooth variable. No interaction term was included. Coefficients in all Poisson or logistic regressions were considered significant at p = 0.05 (R, version 3.1.0, The R Project for Statistical Computing).

## Results

### Descriptive Analyses

Eighteen tick species were identified (N = 14,369) through passive tick surveillance in Ontario (2008–2012) (Table 1). *Ixodes scapularis*, *D*. *variabilis*, *I*. *cookei* and *A*. *americanum* represented 98.1% of all ticks submitted. Of the 18 species, eight were adventive species: *A*. *americanum, Amblyomma cajennense*, *Amblyomma inornatum*, *Amblyomma maculatum*, *Dermacentor andersoni*, *Haemaphysalis punctata*, *Ixodes pacificus* and *Ixodes ricinus*. Travel histories for most of those submitting adventive ticks were not available; however, for those that were available, the location the submitter visited was within the range of the tick species submitted, for example: *A*. *americanum* (submitters reported travel to Florida, Maryland, New Jersey, North Carolina, South Carolina, Texas and Virginia, USA; Mexico), *A*. *cajennense* (Belize, Brazil, Jamaica), *A*. *inornatum* (Florida, USA), *D*. *andersoni* (Calgary, Alberta, Canada), *H*. *punctata* (Kazakhstan), *I*. *pacificus* (California, USA) and *I*. *ricinus* (Portugal, Scotland).

**Table 1:** Ticks specimens submitted for identification in Ontario, Canada (2008–2012).

Tick species	Total ticks (% total per year)					Total (avg %)
	2008	2009	2010	2011	2012	
*Ixodes scapularis*	1,480 (44.0)	580 (53.3)	956 (48.9)	2,291 (65.2)	2,535 (57.0)	7,842 (54.6)
*Dermacentor variabilis*	1,520 (45.2)	384 (35.3)	773 (39.5)	902 (25.7)	1,538 (34.6)	5,117 (35.6)
*Ixodes cookei*	219 (6.5)	60 (5.5)	140 (7.2)	219 (6.2)	200 (4.5)	838 (5.8)
*Amblyomma americanum* *	84 (2.5)	44 (4.0)	38 (1.9)	54 (1.5)	83 (1.9)	303 (2.1)
*Ixodes marxi*	20 (0.6)	6 (0.6)	22 (1.1)	22 (0.6)	32 (0.7)	102 (0.7)
*Amblyomma cajennense* *	10 (0.3)	4 (0.4)	11 (0.6)	9 (0.3)	20 (0.4)	54 (0.4)
*Rhipicephalus sanguineus*	16 (0.5)	5 (0.5)	3 (0.2)	4 (0.1)	8 (0.2)	36 (0.3)
*Dermacentor albipictus*	4 (0.1)	3 (0.3)	6 (0.3)	8 (0.2)	4 (0.1)	25 (0.2)
*Ixodes muris*	2 (0.1)	0 (0.0)	0 (0.0)	0 (0.0)	19 (0.4)	21 (0.1)
*Amblyomma maculatum* *	1 (<0.1)	0 (0.0)	2 (0.1)	1 (<0.1)	7 (0.2)	11 (0.1)
*Dermacentor andersoni* *	0 (0.0)	1 (0.1)	2 (0.1)	1 (<0.1)	1 (<0.1)	5 (<0.1)
*Haemaphysalis leporispalustris*	4 (0.1)	1 (0.1)	0 (0.0)	0 (0.0)	0 (0.0)	5 (<0.1)
*Ixodes pacificus* *	0 (0.0)	0 (0.0)	1 (0.1)	0 (<0.1)	2 (<0.1)	3 (<0.1)
*Ixodes ricinus* *	2 (0.1)	0 (0.0)	0 (0.0)	1 (<0.1)	0 (0.0)	3 (<0.1)
*Amblyomma inornatum* *	0 (0.0)	0 (0.0)	1 (0.1)	0 (0.0)	0 (0.0)	1 (<0.1)
*Haemaphysalis punctata* *	0 (0.0)	0 (0.0)	0 (0.0)	1 (<0.1)	0 (0.0)	1 (<0.1)
*Ixodes dentatus*	0 (0.0)	1 (0.1)	0 (0.0)	0 (0.0)	0 (0.0)	1 (<0.1)
*Ixodes texanus*	0 (0.0)	0 (0.0)	0 (0.0)	1 (<0.1)	0 (0.0)	1 (<0.1)
**Total (%)**	3,362 (100)	1,089 (100)	1,955 (100)	3,514 (100)	4,449 (100)	14,369 (100)

*Adventive species.

The age of submitter was reported in 67.5% (9,697/14,369) of submissions; sex was reported in 63.5% (9,126/14,369) of submissions. Among the four most frequently submitted tick species, the age of submitters was significantly different among all species, with the highest age reported in those submitting *A*. *americanum* (47.0±1.46 y), followed by *I*. *scapularis* (41.0±0.35 y), *D*. *variabilis* (36.3±0.41 y) and *I*. *cookei* (29.7±1.08 y) (p<0.0001) (Table 2). Men submitted significantly more *I*. *scapularis* and *D*. *variabilis* (p<0.0001) compared with women. There was no significant difference according to sex for submissions of *I*. *cookei* and *A*. *americanum* (p>0.05).

**Table 2:** Summary of age and sex of persons submitting selected tick species in Ontario, Canada (2008–2012).

Tick species	Submitter demographics			
	Mean ± SE (range) years old*	Total men	Total women	*X* ^2^ (p-value)†
*Amblyomma americanum*	47.0±1.46a (<1–100)	121	139	1.2 (0.26)
*Ixodes scapularis*	41.0±0.35b (<1–105)	2,958	2,354	68.7 (<0.0001)
*Dermacentor variabilis*	36.3±0.41c (<1–107)	2,184	1,902	19.5 (<0.0001)
*Ixodes cookei*	29.7±1.08d (<1–92)	311	306	0.04 (0.84)

*Means for age of submitters followed by different letters are significantly different at a familywise error rate of p<0.05 (F_3,9462_ = 60.2; p<0.0001).

† Chi-square analyses performed to test null hypothesis that there is no significant difference between the expected and observed numbers of men and women submitting a tick species.

Total submissions include instances where a single person submitted multiple ticks at the same time (n = 256 submitters); 2.3% of tick submissions were part of a multiple-tick submission. Of the 256 persons that submitted multiple ticks at the same time, 61.7% submitted multiple *D. variabilis*, followed by *I*. *scapularis* (28.5%), *I*. *cookei* (3.9%), *R*. *sanguineus* (2.3%), *A*. *americanum* (1.2%), *I*. *marxi* (0.8%), *Ixodes* sp. (0.8%), *D*. *albipictus* (0.4%) and *I*. *muris* (0.4%). The mean number of ticks submitted per person was 1.03±0.002 (range: 1–5 ticks).

Submissions for *I*. *scapularis* adults peaked in May and November; *D*. *variabilis* peaked in June; *I*. *cookei* peaked in June/July/August; and *A*. *americanum* peaked in June (Figure 1). Nymphs of most species were not submitted in sufficient numbers to detect seasonality; however, blacklegged tick nymphs were submitted most often from June through August (86.8%).

**Figure 2:**
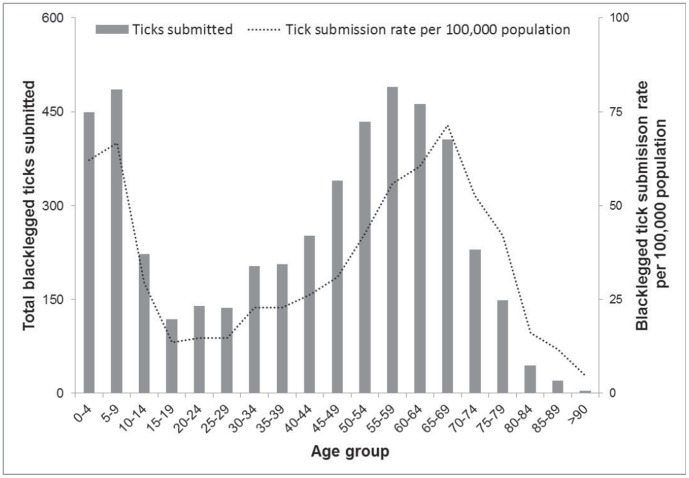
Age distribution of *Ixodes scapularis* submitters, counts and rates per age group, Ontario, Canada (2008–2012). * *For blacklegged ticks submitted in which age was reported by submitter (n = 4,793).

The age distribution of persons submitting blacklegged ticks (total counts) peaked in those less than 10 y and those 45–69 y (Figure 2). Rates of submission per 100,000 population (for each age group) showed that peaks in tick submissions occurred in those less than 10 y and those 55–74 y, with the lowest rates in those 15–29 y. The counts of blacklegged ticks submitted across age groups was moderately independent of the provincial population numbers across age groups (r = 0.50).

The proportion of blacklegged ticks infected with *B*. *burgdorferi* was greatest in adult ticks (15.1%) followed by nymphs (11.3%) (Table 3). Of the blacklegged ticks that were blood-engorged and attached, 7.7% were *B*. *burgdorferi* positive. Of the unengorged and attached ticks, 18.9% were *B*. *burgdorferi* positive. Approximately 65% of the blacklegged ticks tested were attached to the submitter but had not blood fed. The age of submitters of blacklegged ticks that were engorged (45.2±0.80 y) was significantly higher than those submitting blacklegged ticks that were unengorged (40.5±0.54 y) (F_1,2790_ = 24.6; p<0.0001).

**Table 3:** Percentage *Borrelia burgdorferi*-positive *Ixodes scapularis* submitted, based on engorgement and attachment status, in Ontario, Canada (2008–2012).

Engorgement and attachment status*	Total positive pools/total pools tested (%)		Total (average %)
	Adults	Nymphs	
Engorged + attached	146/1,991 (7.3)	18/130 (13.8)	164/2,121 (7.7)
Unengorged + attached	718/3,712 (19.3)	14/153 (9.2)	732/3,865 (18.9)
Unengorged + unattached	9/60 (15.0)	NT†	9/60 (15.0)
**Total (average %)**	873/5,763 (15.1)	32/283 (11.3)	905/6,046 (15.0)

*Engorged ticks were slightly engorged, partially or fully engorged.

† NT, no ticks were tested in this category.

For all blacklegged ticks submitted, 93.4% were adult females, 4.7% were nymphs, 1.7% were adult males and 0.2% were larvae. Of the 288 blacklegged tick nymphs submitted 80.0% were submitted from health units along the north shore of Lake Ontario/St. Lawrence River (EOH, HPE, KFL, LGL) and 6.9% were submitted from health units along the north shore of Lake Erie (CHK, ELG, HDN, NIA, WEC).

Based on rates of submission per 100,000 population, submitters from KFL and LGL were 54× more likely to encounter a blacklegged tick, 45× more likely to detect a blood-feeding blacklegged tick and 47× more likely to have an infected, blood-feeding blacklegged tick on them compared with submitters from the rest of province (Table 4). The health units of KFL and LGL contain 2.8% of the province's total population yet contributed to over 57% of all infected, blood-fed blacklegged ticks.

**Table 4:** *Ixodes scapularis* submission rate per 100,000 population, based on attachment status, level of engorgement and infection rate with *Borrelia burgdorferi* in ticks collected by passive surveillance in Ontario, Canada (2008–2012).

Status of submitted blacklegged ticks	Submission rate per 100,000 population*	
	LGL & KFL health units†	Remainder of Ontario (all Ontario)
Attached	1021.7	18.9 (46.9)
Attached + engorged‡	335.4	7.4 (16.6)
Attached + engorged + infected with *Borrelia burgdorferi*	26.5	0.56 (1.3)

*Ticks submissions based on place of exposure as reported by submitter.

† LGL: Leeds, Grenville & Lanark District; KFL: Kingston-Frontenac and Lennox & Addington.

‡ See Table 3 for definitions of engorgement.

### Mapping

**Figure 3:**
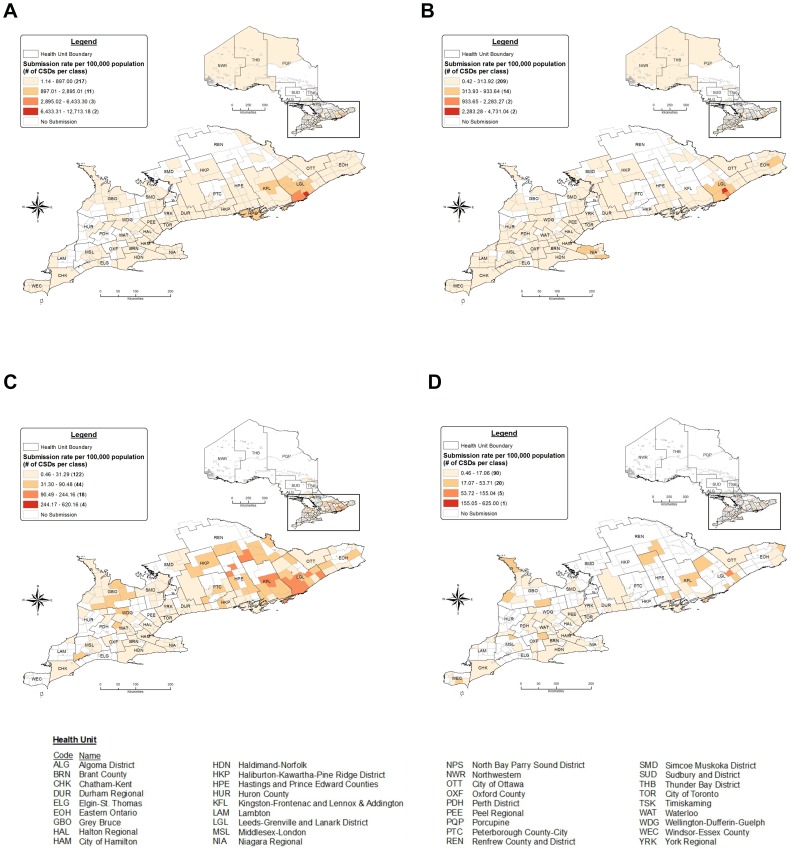
Distribution of ticks submitted for identification in Ontario, Canada (2008–2012) as rates of submission per 100,000 population (based on submitter CSD of residence). A) *Ixodes scapularis*, B) *Dermacentor variabilis*, C) *Ixodes cookei* and D) *Amblyomma americanum*.† †CSD, census subdivision. All CSDs not visible because of their small size.

For submitters, 92.6% (13,299/14,369) of submissions reported locality or town of residence and, for blacklegged ticks, 97.0% (5,962/6,147) of submissions reported town of blacklegged tick acquisition. Most tick submissions (96%) in Ontario are from the southern portion of the province (south of 46°N). *Ixodes scapularis* were submitted at higher rates per 100,000 population from CSDs located in HPE, KFL and LGL health units in Ontario's Eastern region (Figure 3A). Higher rates of submission for *D. variabilis* were noted in NIA and LGL, from the Central West and Eastern regions, respectively (Figure 3B). The highest rates of submission for *I*. *cookei* were reported from HPE, KFL, LGL and REN in the Eastern region and GBO in the South West region (Figure 3C). The highest rates of *A. americanum* submissions were from LGL in the Eastern region (Figure 3D). In addition, we present tick distributions as point maps with tick relative abundance (Figure S2).

**Figure 4:**
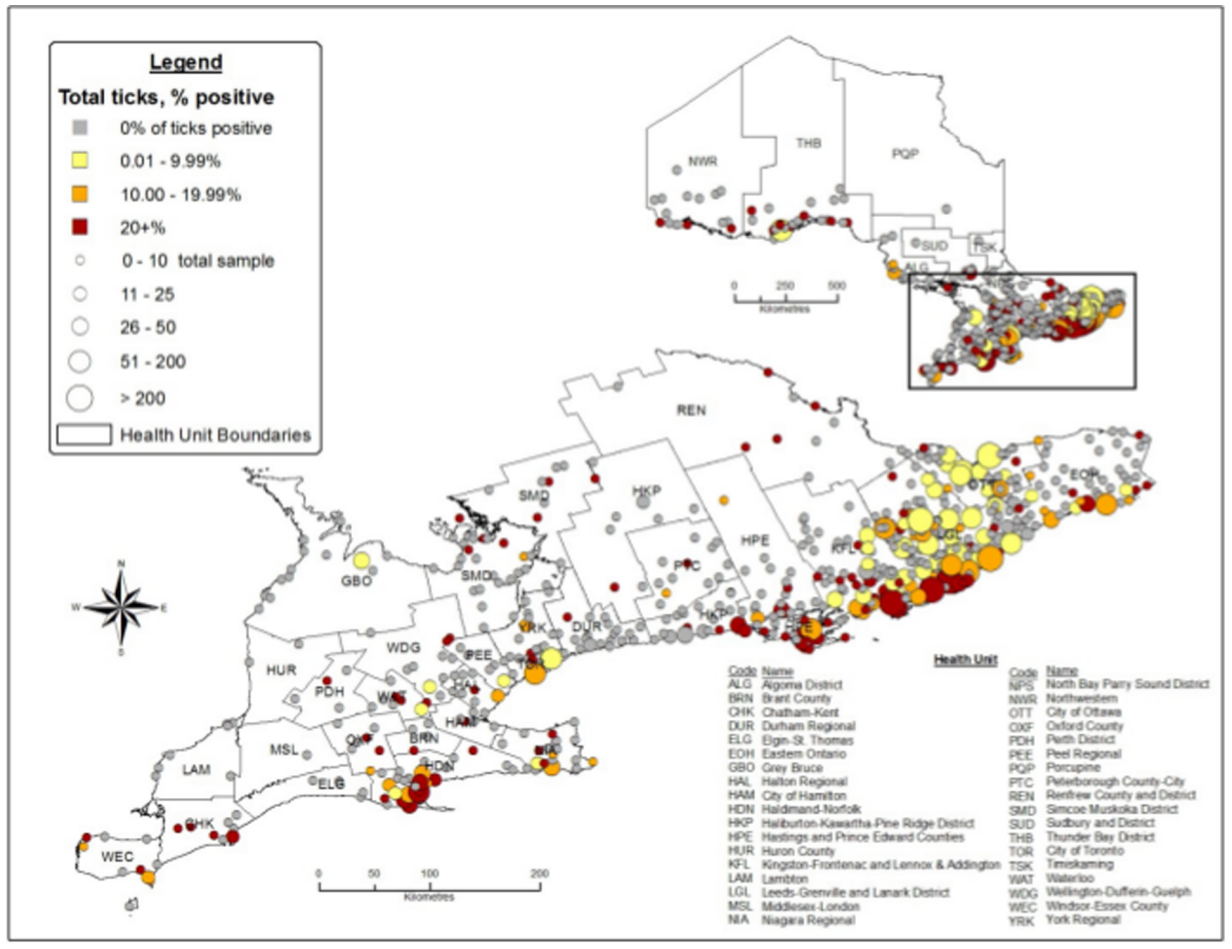
Percentage *Borrelia burgdorferi*-positivity and total *Ixodes scapularis* by submitter town of exposure in Ontario, Canada (2008–2012).

Localities with the highest percent (>20%) *B*. *burgdorferi*-positive blacklegged ticks and highest numbers of blacklegged ticks submitted were in the Eastern region (HPE, KFL, LGL) and to a lesser extent in the Central West region (HDN) (Figure 4).

### Multivariable Analyses

**Figure 5:**
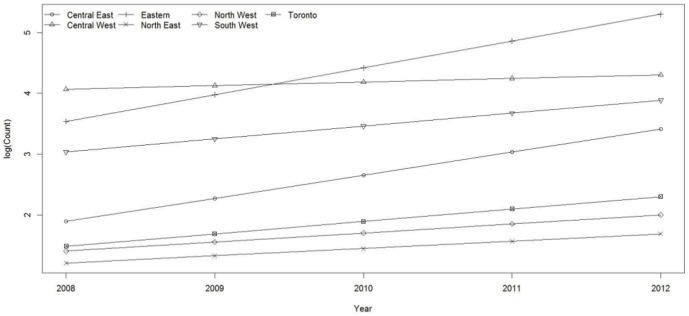
Trends in *Ixodes scapularis* counts in Ontario, Canada, by region (2008–2012).

Overall counts (GAM) of blacklegged ticks were highest in the Eastern region followed by Central West and South West region; the lowest counts were from the North East region, followed by North West, Toronto and Central West regions (Figure 5). The rate of increase (slope) in counts of blacklegged tick submissions was greater in the Eastern and Central East regions, with all other regions with lower rates of increase. In Eastern and Central East regions, there are significant increases in blacklegged ticks submissions over the surveillance period and this increase is significantly greater than all other regions (p<0.0001) (Figure 5).

**Figure 6:**
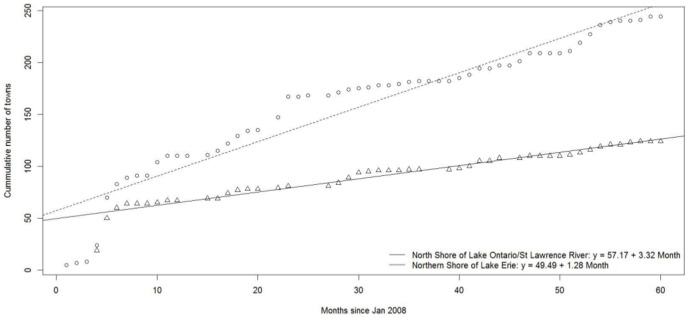
Rate of spread of *Ixodes scapularis*, measured in cumulative number towns with blacklegged ticks submissions along the northern shores of Lake Ontario/St. Lawrence River and Lake Erie, Ontario, Canada (2008–2012). * *Includes *I*. *scapularis* submitted from: human (n = 6,147), dog (1,961), cat (206), environment (24), white-tailed deer (6), horse (3) and other animals not specified or unknown (51).

In examining the rate of spread of blacklegged ticks over time (y = 49.49+1.28 Month+7.68 Region+2.04 Month*Region; R^2^ = 0.94), we found that spread along the north shore of Lake Ontario/St. Lawrence River was relatively faster than in health units along the northern shore of Lake Erie (2.04/month, p<0.0001) (Figure 6).

Provincially, the percent of blacklegged ticks infected with *B*. *burgdorferi* increased over time, from 8.4% in 2008, 2009 (11.3%), 2010 (12.2%), 2011 (15.1%) to 19.1% in 2012 (overall average = 15.0%), with an increase of 2.5% per year. The odds of *B. burgdorferi*-positive blacklegged tick submissions increased each year (OR = 1.34; p<0.0001) and was greatest in the Central West region (OR = 2.34; p = 0.00013), followed by the Eastern region (OR = 1.79; p = 0.0031) and South West region (OR = 2.05; p = 0.014) compared to the North East region (reference region).

Provincially, the percent of blacklegged ticks infected with *A*. *phagocytophilum* ranged from 0.30% in 2008, 2009 (0.50%), 2010 (0.39%), 2011 (0.31%) to 0.32% in 2012 (overall average = 0.30%). The number of *A. phagocytophilum*-positive blacklegged tick submissions (modelled using logistic regression) did not display any temporally or spatially significant trends. *Anaplasma phagocytophilum* was detected primarily (11 out of 21) in blacklegged ticks submitted from Ontario's Eastern region (KFL, LGL, OTT).

## Discussion

*Ixodes scapularis* was the most abundant tick submitted by the Ontario public starting in 2009, followed by *D*. *variabilis*, *I*. *cookei* and *A*. *americanum*. In Maine (USA), *I*. *scapularis*, *D*. *variabilis*, *I*. *cookei* and *D*. *albipictus* were the most abundant species detected during passive surveillance [20]. In Michigan (USA), the top four species were *D*. *variabilis*, *D*. *albipictus*, *I*. *cookei* and *I*. *scapularis* [21]. Differences in species composition and relative abundance between the current study and previous studies are because of variable climates and habitats, differences in surveillance periods and differences in how ticks were submitted (e.g., deer-derived ticks included in Michigan study). Ontario's 2010 public education campaign (*Let*'*s Target Lyme*) heightened tick awareness throughout the province and possibly increased submissions for all tick species, especially in health units with historical blacklegged tick populations [2], [22]. The Eastern region of Ontario (KFL, LGL) reported the highest submission rates for not only *I*. *scapularis* but also for *D. variabilis* and *I. cookei*. In 2009, blacklegged ticks became the most common tick submitted in Ontario and, based on this study and others [4], [6], encounters with blacklegged ticks will continue to increase in frequency as their populations expand into new localities.

Following *I*. *scapularis* in relative abundance, *D*. *variabilis*, *I*. *cookei* and *A*. *americanum* are the most frequently submitted tick species in Ontario. In North America, contiguous populations of *D*. *variabilis* are typically restricted to south of the 0°C midwinter isotherm (southern Ohio), except in northern areas where large bodies of water such as the Great Lakes moderate cool temperatures and allow for survival of this species [23], [24]. In Ontario, *D*. *variabilis* was most often reported from towns along major bodies of water in the South West (shores of Lake Huron, Lake St. Clair, Detroit River), Central West (shores of Lake Ontario, Niagara River) and Eastern regions (northern shore of St. Lawrence River). Based on the presence of established populations, submission rates of American dog ticks were lower than expected (possibly because of public familiarity with the species) in health units such as BRN, CHK, LAM, NWR, WEC (LRL unpublished data). American dog ticks infected with *Francisella tularensis* (agent of tularemia) and *Rickettsia rickettsii* (Rocky Mountain spotted fever) have been detected in Ontario [25], [26]. *Ixodes cookei* was more commonly submitted from the South West and Eastern regions of Ontario, particularly in areas dominated by agricultural lands and mixed wood forests, areas favorable for one of the ticks important hosts, the groundhog *Marmota monax* [15], [27]. *Ixodes cookei* is a vector of Powassan encephalitis virus (POWV); POWV was isolated from *I*. *cookei* in the province in the late 1970s [26]. Further research is required to determine whether Lineage I strains of POWV (also referred to as deer tick virus) are infecting blacklegged ticks in Ontario and elsewhere in Canada [28]. Maintaining an understanding of the distribution of ticks will assist in risk assessments for tick-borne pathogens such as POWV and *R. rickettsii*.

Eight tick species identified in Ontario were adventive species, presumably as immigrants to Ontario via human travel or introduced on migratory birds or other wildlife. The probability of these immigrant species, apart from the lone star tick *A*. *americanum*, to establish in Ontario would be considered unlikely given their rarity (i.e., *I*. *ricinus*, *H*. *punctata*) and the lack of suitable climate or hosts (i.e., *A*. *cajennense*, *A*. *maculatum*). The scattered distribution of the lone star tick, the limited multiple specimens per submitter and the relatively small numbers encountered suggest that *A*. *americanum* is not yet established in Ontario. Active surveillance involving tick drags throughout the province since 2008 have not detected *A*. *americanum* populations, but small numbers of specimens (one per locality) have been encountered when drag sampling at Rondeau Provincial Park and St. Lawrence Islands National Park (CR, LRL unpublished data). The scarcity of the species during active surveillance is consistent with the hypothesis that occasional introductions of lone star ticks occur in Ontario but populations have not taken up residence. The range of *A*. *americanum* has expanded into northern and western parts of New York State (1988–1996), including counties bordering Ontario [29]. In Michigan, *A*. *americanum* was the fifth most submitted tick, yet they were not routinely collected in the state's active tick surveillance [21]. As climate change has contributed to the spread of blacklegged ticks into southern Canada, a similar fate could occur with *A*. *americanum* in the coming decades [6], [30]. *Amblyomma americanum* is a vector of several human pathogens, including *Ehrlichia chaffeensis* (agent of human ehrlichiosis) [31]. Passive tick surveillance affords public health with the capacity to detect incipient invasions of adventive tick species and, when indicated, their associated pathogens.

Children 0–9 years old and adults 55–74 years old have the highest submission rates for blacklegged ticks in Ontario. Although rates of submission were not uniform across all age groups, it is unknown whether this is caused by differences in exposure to tick habitats, host behaviors or tick submission efforts. A case-control study in Connecticut (USA) noted that frequent outdoor activity was not associated with increased risk of Lyme disease; however, it is not clear that these results would also apply to tick exposure [32]. Men submit more blacklegged ticks in Ontario than women do, which is expected because women are more likely to avoid tick-infested areas [33]. Men also submitted more *D. variabilis* than women (no sex difference for *A. americanum* and *A. americanum*); however, the underlying biological reasons for sex-based submissions of various tick species remains to be tested. Submission counts among age groups were moderately independent of the population for each age group, revealing that submission effort alone among the age groups cannot explain higher or lower blacklegged tick submissions (i.e. exposure among age groups varies). Higher numbers of submitters in young age groups was noted in Maine (39% of submissions from 0–14 year olds); the relatively higher proportion of ticks from this age group was putatively caused by increased attention to ticks on children during parental care [20], [21]. Higher rates of tick submissions from children are a concern given that the age distribution of Lyme disease cases in the USA follows a similar pattern where higher case counts occur in children and older adults [34]. In contrast, the pattern in Ontario does not show a peak in children cases, rather a single peak in those aged 50–59 years old (unpublished data). Approximately 33% of submissions did not include age of submitter and 37% were missing sex; however, there is no indication to suggest that cases with missing age or sex are appreciably different from the trends reported here. Engorged blacklegged ticks are more likely to transmit *B. burgdorferi*; therefore, the higher mean age noted for those submitting engorged ticks (45.2 years old) compared with unengorged ticks (40.5 years old) indicates that public education surrounding the early detection and removal of ticks should focus on the older portion of the population. Maine's passive surveillance noted an increase in engorged blacklegged tick submission with age, attributed to decreasing visual acuity with increasing age [20]. The relatively higher rate of tick submissions in children and older adults (plus lower rates of submission in 15–29 year olds) requires additional research to identify behavioral or ecological factors contributing to variable exposure to ticks. Additionally, the higher incidence of ticks found on these two age groups represents an opportunity for focused education programs on personal protective measures.

The proportion of blacklegged ticks infected with *B*. *burgdorferi* has increased in Ontario from 2008 through 2012, particularly in the Central West, Eastern and South West regions. Given the time lag between tick invasion and establishment, we expect a continued increase in *B. burgdorferi* positivity among reservoir hosts and tick populations [12]. Although the percentage of ticks infected with *B*. *burgdorferi* is increasing, it will reach a stable but fluctuating higher overall prevalence in 5 to 7 years [35]. The overall prevalence of *B*. *burgdorferi* infection in Ontario blacklegged ticks was 15%, similar to other emerging areas such as Manitoba (10%), Quebec (13%) or Nova Scotia (15%), but much lower than in long-established areas such as the Hudson Valley, New York (49–65%), New Jersey (49%) or even the historical population at Long Point, Ontario (67%) [6], [13], [36]–[38]. The higher percent of positive blacklegged ticks (18.9%) that were unengorged and not attached—compared with engorged and attached (7.7%)—is not in agreement with recent work in Canada. For example, in Quebec, engorged blacklegged ticks had a higher percent *B*. *burgdorferi*-positivity (10.7%) than unengorged ticks (5.9%); from across Canada, engorged blacklegged ticks had a higher percent *B*. *burgdorferi*-positivity (13.2%) than unengorged ticks (9.2%) [12], [13]. However, recent work in Canada agrees with our findings where *B. burgdorferi* detection was more likely from unfed ticks compared to slightly, partially or fully engorged ticks [39]. Multiplication of *B. burgdorferi* spirochetes has been reported to increase in blood-feeding blacklegged ticks by 300×, at least in nymphal stage; however, in our study, most engorged ticks were adults, meaning multiplication of spirochetes may not occur in adults as they do in nymphs, resulting in our lower infection rates for engorged ticks [40]. The percentage *B*. *burgdorferi*-positive blacklegged ticks will continue to increase in Ontario as tick populations expand.

Less than 5% of all blacklegged tick submissions in Ontario (2008–2012) were nymphs. Although the percentage of nymphs is relatively low, it is higher than previous reports for passive tick surveillance across Canada (1990–2003), where nymphs represented only 1% of all submissions [13]. In Maine, nymph submissions increased over time and correlated with an increased incidence of Lyme disease [20]. The relatively low numbers of nymphs submitted in Ontario is partly because of their small size, but we expect submissions to increase as the public and health care professionals become more proficient at detecting them. The low numbers of nymph submissions is also a function of the relatively recent establishment of blacklegged tick populations in Ontario, as is the case for other regions of Canada [30]. Our results indicate that nymph submissions continue to increase in Ontario, further increasing risk of *B*. *burgdorferi* infection.

The rate of blacklegged tick spread is fastest in the Eastern region of Ontario, a region that includes health units with blacklegged tick (engorged and *B*. *burgdorferi* positive) submission rates 47× higher than the rest of the province. The areas in the Eastern region of Ontario with the highest percentage *B*. *burgdorferi*-positive ticks (and highest numbers of blacklegged tick submission) coincide with areas where there is increased incidence of Lyme disease [41]. Although long-established blacklegged tick populations exist along the northern shore of Lake Erie, the spread of blacklegged ticks from these isolated populations was slower than spread from established populations along the north shore of Lake Ontario/St. Lawrence River. We hypothesize that the measured spread from established populations along the north shore of Lake Erie is caused by reduced suitable habitat for ticks and hosts (i.e., deciduous forest) [9]. The north shore along Lake Erie has a higher percentage of agricultural land (67%) and a lower percentage of forested land (8.5%); compared with the north shore of Lake Ontario/St. Lawrence River with 56% agricultural land and 17% forested land [15]. If temperature alone was responsible for spread of blacklegged ticks in Ontario, we would expect populations along the north shore of Lake Erie to spread faster since they are located in the warmest regions of the province (i.e., >3400 annual degree days above 0°C) [42]. Generally, temperature is the driving factor for northward spread of the blacklegged tick in North America; however, other factors are essential for spread at the regional level, such as availability of suitable habitat and hosts [3], [6]. Our estimates for spread of blacklegged tick populations are in agreement with recent climatic models of population expansion in southern Ontario, where expansion is faster in the Eastern region of the province [4]. Blacklegged tick populations have expanded rapidly in the Eastern region where populations are essentially contiguous or at least composed of numerous (greater than current number of two) established populations.

Passive surveillance data are consistent with more established blacklegged tick populations exist in the Eastern region of the province (e.g., higher submission rates in multiple CSDs, higher rates of spread, increased nymph submissions). Outside of the Eastern region of the province, except along the north shore of Lake Erie, we hypothesize that detections of blacklegged ticks represent adventive individuals via bird-borne introductions [13]. Although passive surveillance is useful in detecting blacklegged tick populations in the Eastern or Central/South West regions, the probability of detecting populations outside of these regions is less reliable because of a lower probability of the public encountering ticks over a large geographic area. Where human population density is low (North West region = 1.7 persons/km^2^; rest of Ontario = 37.0 persons/km^2^), we suspect passive surveillance will be unsuccessful in the detection of established blacklegged tick populations and, in these cases, active surveillance (live-mammal trapping) will remain the gold standard [14]. Initiation of active surveillance in sparsely populated areas will rely on human case surveillance accompanied by accurate exposure information. The costs and benefits of active surveillance versus passive surveillance need to be assessed when identifying locales where the public is at greatest risk for Lyme disease.

Work describing the distribution of Ontario tick species is scattered, but include Bequaert (1945), Wilkinson (1967) and Gregson (1956) [43]–[45]. Although these works give a broad picture of ticks in Ontario, they lack the details needed to define current tick distribution and potential human, tick-borne disease risk. Although active surveillance for ticks remains the gold standard for determining tick distribution and relative abundance, our research indicates that passive tick surveillance remains an effective tool. The demographics of those submitting ticks indicate that men were more likely to submit blacklegged ticks than women and more blacklegged ticks were being submitted from younger children and older adults, representing portions of the population requiring focused public education on ticks and tick-borne disease. The Eastern region of Ontario is the primary area of blacklegged tick submissions and *B*. *burgdorferi*-positivity in the province and an area where tick populations are developing over a broad geographic area and not in a focal manner, as has been the situation with the other historical tick populations. As climate change increases the suitability of Ontario for the establishment of adventive species and the expansion of blacklegged tick populations, passive tick surveillance will play a key role in detecting tick populations that pose a threat to public health.

## Supporting Information

Figure S1 Ontario's public health units and health regions.
(TIF)

Figure S2 Submitter town of residence for ticks in Ontario, Canada (2008–2012). A) *Ixodes scapularis*, B) *Dermacentor variabilis*, C) *Ixodes cookei* and D) *Amblyomma americanum*.
(TIF)
