# Supplementary figures and images for: Population-Based Passive Tick Surveillance and Detection of Expanding Foci of Blacklegged Ticks Ixodes scapularis and the Lyme Disease Agent Borrelia burgdorferi in Ontario, Canada
Source: PLoS One. 2014 Aug 29;9(8):e105358. doi: 10.1371/journal.pone.0105358 (PMC4149368; doi:10.1371/journal.pone.0105358)

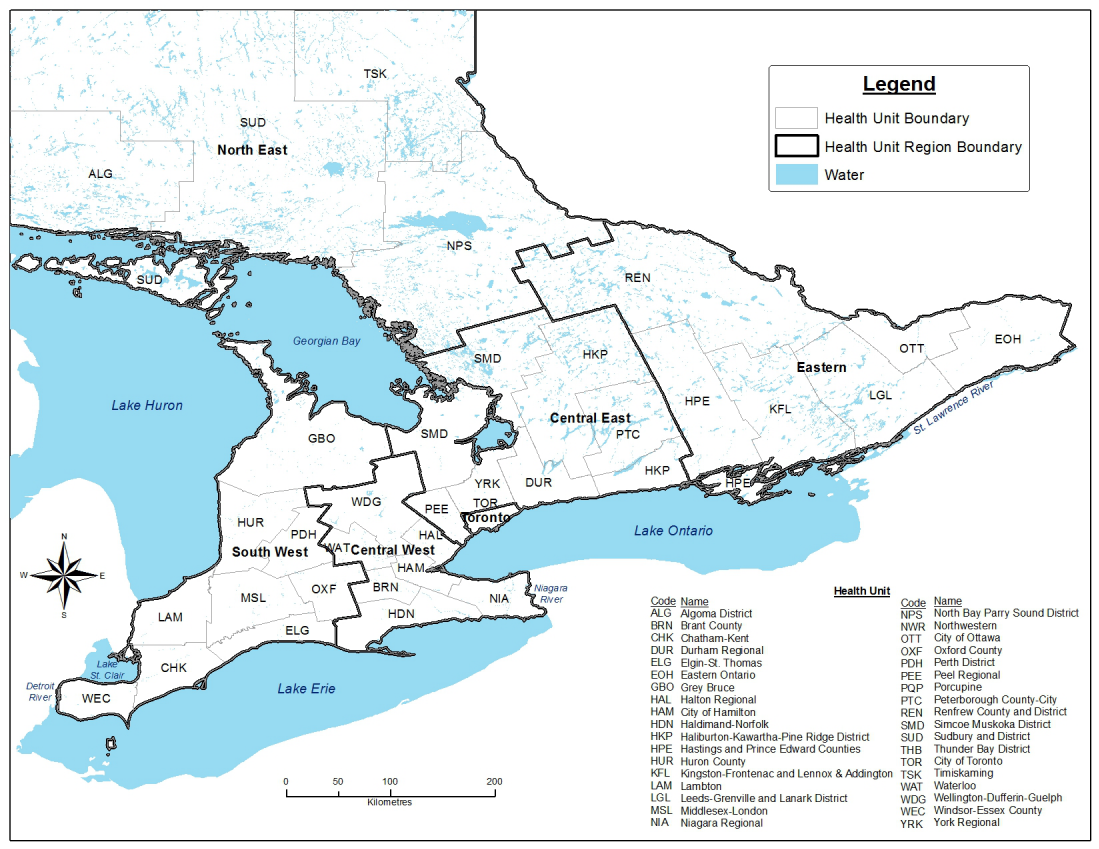

Supplement: Figure S1 — Ontario's public health units and health regions. (TIF) [file pone.0105358.s001.tif]

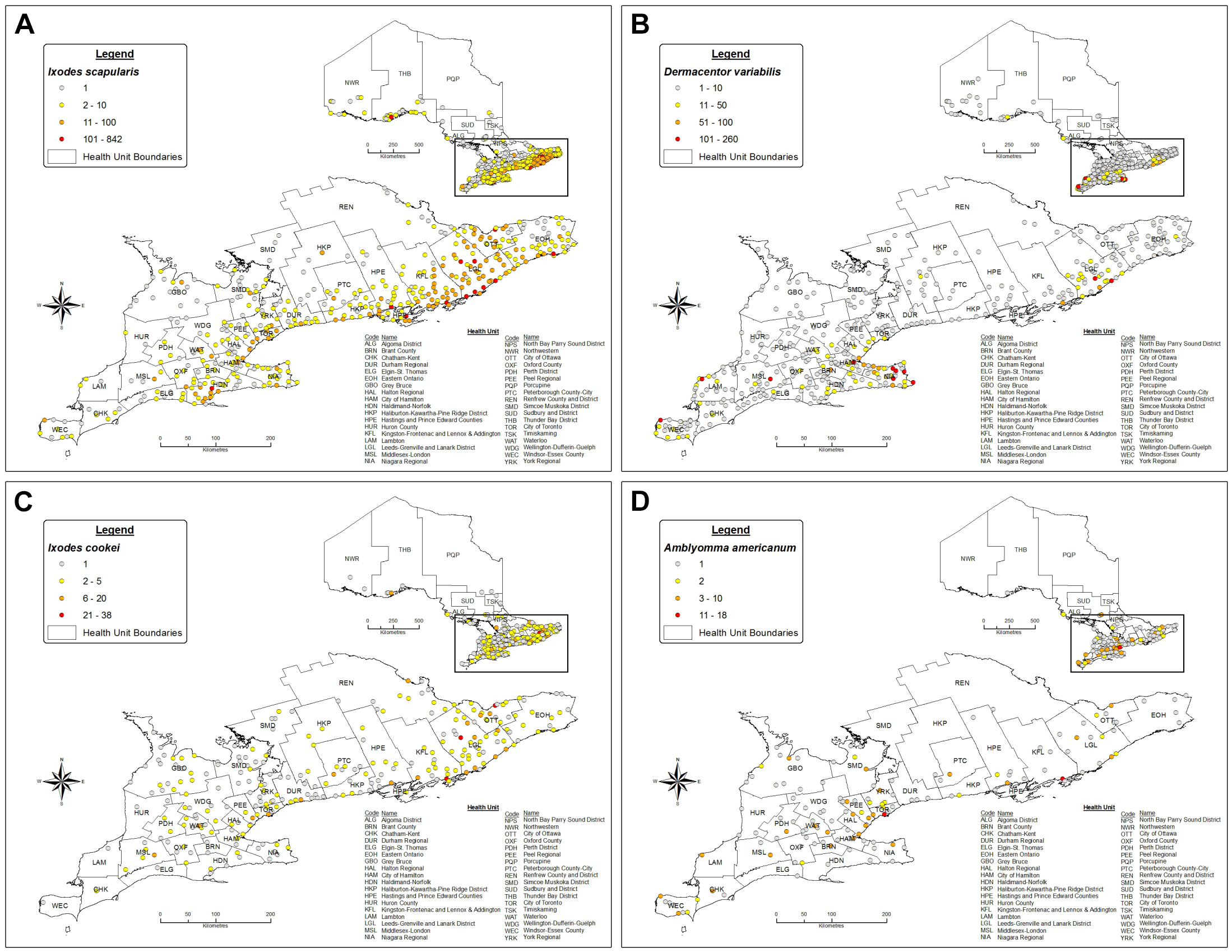

Supplement: Figure S2 — Submitter town of residence for ticks in Ontario, Canada (2008–2012). A) Ixodes scapularis, B) Dermacentor variabilis, C) Ixodes cookei and D) Amblyomma americanum. (TIF) [file pone.0105358.s002.tif]
